# Effects of Lenvatinib combined with immune checkpoint inhibitors on efficacy, immune function and prognosis in patients with primary liver cancer

**DOI:** 10.12669/pjms.41.12.12763

**Published:** 2025-12

**Authors:** Yuehong Hou, Zhenlin Gao, Ci Liu, Yajing Zhang, Dongliang Zhang

**Affiliations:** 1 Yuehong Hou, Department of Medical Oncology, Shijiazhuang People’s Hospital, Shijiazhuang 050000, Hebei, China; 2 Zhenlin Gao, Department of Medical Oncology, Shijiazhuang People’s Hospital, Shijiazhuang 050000, Hebei, China; 3 Ci Liu, Department of Medical Oncology, Beijing Water Conservancy Hospital, Beijing 100000, Beijing, China; 4 Yajing Zhang, Department of Medical Oncology, Shijiazhuang People’s Hospital, Shijiazhuang 050000, Hebei, China; 5 Dongliang Zhang, Department of Neurosurgery, Shijiazhuang People’s Hospital, Shijiazhuang 050000, Hebei, China

**Keywords:** Efficacy, Immune checkpoint inhibitors, Immune Function, Lenvatinib, Primary liver cancer

## Abstract

**Objective:**

To evaluate the clinical efficacy, immune function effects and safety of lenvatinib combined with immune checkpoint inhibitors (sintilimab) in treating advanced primary liver cancer.

**Methodology:**

A retrospective cohort study was conducted on 80 patients with advanced liver cancer in Shijiazhuang People’s Hospital from January 2023 to June 2024, who were divided into an observation group (lenvatinib + sintilimab, n=40) and a control group (lenvatinib monotherapy, n=40). Specifically, the overall response rate (ORR), immune function indicators (CD3+, CD4+, CD4+/CD8+, IgM/IgG/IgA), quality of life (SGRQ scores) and adverse reactions were compared between the two groups.

**Results:**

The ORR in the observation group (72.5%) was significantly higher than that in the control group (50.0%), with statistically significant differences (*P*=0.039). After treatment, the improvement in levels of CD3+, CD4+, CD4+/CD8+ and immunoglobulins (IgM, IgG, IgA) in the observation group significantly outperformed those in the control group, with statistically significant differences (all P<0.05). Additionally, the observation group reported more substantially improved SGRQ scores than the control group and differences were statistically significant (*P*<0.001). In terms of safety, no statistical differences were observed regarding the incidence of adverse reactions in both groups (*P*=0.369).

**Conclusion:**

Lenvatinib combined with immune checkpoint inhibitors can significantly improve the objective response rate in patients with advanced liver cancer, enhance their immune function and improve their quality of life, demonstrating satisfactory safety.

## INTRODUCTION

Primary liver cancer is one of the most common malignant tumors clinically and according to the World Health Organization, over 900,000 new cases and about 830,000 deaths of liver cancer were reported annually, with its incidence and mortality ranking sixth and third among malignant tumors, respectively, alongside a five-year survival rate of less than 20% for patients with advanced liver cancer.^1^ Due to the insidious onset of liver cancer, over 60% of patients are diagnosed at advanced stages, losing the opportunity for surgical cure.^2^ Currently, the treatment of advanced liver cancer has evolved into an era of multimodal combination therapy. Lenvatinib is a multi-targeted tyrosine kinase inhibitor that significantly inhibits tumor angiogenesis and tumor cell proliferation by targeting signaling pathways such as VEGFR1-3, FGFR1-4, PDGFRα, RET and KIT. Clinical studies have confirmed that lenvatinib shows a significantly better objective response rate and progression-free survival than sorafenib in treating unresectable primary liver cancer.

However, the survival benefits of monotherapy are limited and long-term use can lead to resistance.^3^ By contrast, immune checkpoint inhibitors can inhibit the recognition function of small molecular proteins in immune cells, regulate the level of immune activation and exert antitumor effects. In this regard, the immunosuppressive microenvironment of liver cancer has been found to be highly heterogeneous, which may be a key factor limiting the efficacy of immune checkpoint inhibitors as monotherapy.^4^ Based on the interaction mechanism between tumor angiogenesis and the immunosuppressive microenvironment, the combination strategy of lenvatinib and immune checkpoint inhibitors demonstrates synergistic potential. Research indicates that lenvatinib can reverse immunosuppressive effects by blocking the VEGF pathway, while immune checkpoint inhibitors can further activate tumor-specific T cell responses.^4^,^5^

However, there are still issues to be addressed regarding clinical studies of this combination regimen: firstly, Most studies come with limited sample sizes and short follow-ups; secondly, the impact of combination therapy on the dynamic changes in the immune function of patients has not been fully elucidated; thirdly, There is a lack of reliable predictive biomarkers to screen for the optimal beneficiary population; finally, it is necessary to monitor and assess whether the combination increases the risk of additive toxicity. Given the above, this study intends to systematically evaluate the clinical efficacy and immune function effects of lenvatinib combined with immune checkpoint inhibitors in the treatment of advanced primary liver cancer.

## METHODOLOGY

A retrospective cohort design was adopted in this study, including 80 patients with advanced primary liver cancer who were treated at Shijiazhuang People’s Hospital between from January 2023 to June 2024. Based on the treatment regimen, patients were divided into an observation group (lenvatinib + immune checkpoint inhibitors) and a control group (lenvatinib monotherapy).

### Ethical approval:

The study was approved by the Institutional Ethics Committee of Shijiazhuang People’s Hospital (No.:[2024]003; date: February 05, 2024) and written informed consent was obtained from all participants.

### Inclusion criteria:

- Aged 18-75 years, meeting the diagnostic criteria for primary liver cancer as per the Chinese Society of Clinical Oncology Guidelines (2024 Edition).^3^
- TNM staging II~IV Phase.
- Child-Pugh score ≤ 7 points for liver function.
- ECOG Performance Status score of 0-2 points.
- At least one measurable lesion present.
- No prior systemic anti-tumor treatment or completion of previous treatment at least four weeks prior.
- Expected survival time ≥ 3 months.

### Exclusion criteria:

- Patients combined with other malignancie and patients with metastatic liver cancer.
- Those combined with severe heart, lung, or kidney dysfunction.
- Those with active autoimmune diseases or those requiring long-term immunosuppressive therapy.
- Those with a history of severe allergic reactions to lenvatinib or immune checkpoint inhibitors.
- Pregnant or breastfeeding women.

Patients in the observation group received oral lenvatinib (12 mg/day for those ≥ 60 kg, 8 mg/day for those < 60 kg) once daily, along with intravenous infusions of immune checkpoint inhibitors (sintilimab 200 mg) every three weeks. By contrast, the control group was given only the same dose of lenvatinib monotherapy. Both groups continued treatment until disease progression (PD), intolerable toxicity, or withdrawal from the study.

### Observation indicators:

- *Clinical Efficacy:* Evaluated according to the criteria published by the Union for International Cancer Control (UICC) and the World Health Organization (WHO), efficacy is classified into four levels: Complete Response (CR): All tumor lesions completely disappear, maintained for at least one month; Partial Response (PR): Tumor lesions decrease by > 50% with no disease progression, maintained for at least one month, during which no new lesions appear; Stable Disease (SD): Lesions decrease by < 50% or increase by ≤25%, maintained for at least one month; and Progressive Disease (PD): Tumor lesions increase by > 25% or new lesions appear. Overall Response Rate = (number of CR + number of PR) / total number × 100%^6^.
- *Immune Function:* Fasting in the in the morning, peripheral blood samples were collected from patients at baseline and during the 3rd and 6th cycles of treatment, with flow cytometry used to detect T lymphocyte subsets (CD3+, CD4+, CD4+/CD8+) and an automatic immunoassay analyzer utilized to determine the levels of immunoglobulin M (IgM), immunoglobulin G (IgG) and immunoglobulin A (IgA), followed by an analysis of changes in these indicators pre- and post-treatment.
- *Quality of Life:* The quality of life pre- and post-treatment was assessed using the St. George’s Respiratory Questionnaire (SGRQ), with higher scores indicating worse quality of life.
- *Assessment of Adverse Drug Reactions:* The safety of the treatment regimen was assessed using the National Cancer Institute Common Toxicity Criteria version 3.0 (NCICTC 3.0).

### Statistical analysis:

SPSS 20.0 software was used for the statistical analysis. Count data were expressed as n (%) and inter-group comparisons were conducted using the χ^2^ test. Measurement data were expressed as Mean±SD (χ̄±*S*), with independent sample t-tests performed for inter-group comparisons. *P*<0.05 was considered statistically significant.

## RESULTS

A total of 80 patients with primary liver cancer admitted to Shijiazhuang People’s Hospital from January 2023 to June 2024 were collected and divided into an observation group (n=40) and a control group (n=40), with December 31, 2024 as the follow-up deadline. The observation group: patients aged 43-70 years, with a mean age of 57.60±7.89 years; the control group: patients aged 41-72 years, with a mean age of 58.85±6.55 years. No significant differences were observed in the general data between the two groups, exhibiting the inter-group comparability (Table-I).

**Table-I:** Comparison of General Data between the two Groups.

Indicator	Observation Group	Control Group	t/χ^2^	P
Age (Years)	57.60±7.89	58.85±6.55	0.771	0.443
Gender (M/F)	32/8	30/10	0.287	0.592
Hepatitis			0.771	0.856
No	8 (20.00%)	6 (15.00%)		
B	15 (37.50%)	14 (35.00%)		
C	16 (40.00%)	18 (45.00%)		
B+C	1 (2.50%)	2 (5.00%)		
Cirrhosis			0.474	0.491
Yes	26 (65.00%)	23 (57.50%)		
No	14 (35.00%)	17 (42.50%)		
Child-Pugh Class			0.827	0.363
A	32 (80.00%)	35 (87.50%)		
B	8 (20.00%)	5 (12.50%)		
ECOG PS (points)			2.225	0.329
0	6 (15.00%)	5 (12.50%)		
1	32 (80.00%)	35 (87.50%)		
2	2 (5.00%)	0 (0.00%)		
AFP(ng/ml)	436.90±28.41	434.07±33.02	0.410	0.683
Metastases			0.105	0.745
Yes	6 (15.00%)	5 (12.50%)		
No	34 (85.00%)	35 (87.50%)		
Vascular Invasion			0.228	0.633
Yes	12 (30.00%)	14 (35.00%)		
No	28 (70.00%)	26 (65.00%)		

The overall response rate (ORR) in the observation group (72.50%) was higher than that in the control group (50.00%), with remarkable differences (*P*<0.05) (Table-II).

**Table-II:** Comparison of Clinical Efficacy between the two Groups [n (%)].

Group	CR	PR	SD	PD	ORR
Observation (n=40)	8 (20.00)	21 (52.50)	8 (20.00)	3 (7.50)	29 (72.50)
Control (n=40)	5 (12.50)	15 (37.50)	10 (25.00)	10 (25.00)	20 (50.00)
*χ^2^value*					4.266
*P value*					0.039

Before treatment, no significant differences in CD3 +, CD4 +, CD4 +/CD8 +, IgM, IgG and IgA levels were observed between the two groups (*P*>0.05); after treatment, CD3 +, CD4 +, CD4 +/CD8 +, IgM, IgG and IgA levels in the two groups were significantly improved compared with pre-treatment levels (*P*<0.05), with more substantial improvement seen in the observation group than in the control group and the differences were statistically significant (*P*<0.05) (Table-III).

**Table-III:** Comparison of pre and post-treatment immune function levels between the two groups (*Χ̅±S*).

Observation Indicator	Observed Point	Observation Group	Control Group	t	p
CD3^+^ (%)	Pre-treatment	42.62±6.44	43.00±6.12	0.269	0.789
	Post-treatment	48.42±6.41	43.59±6.12	3.450	0.001
CD4^+^ (%)	Pre-treatment	27.12±4.34	27.51±3.83	0.421	0.675
	Post-treatment	37.97±5.12	34.15±4.85	3.432	0.001
CD4^+^/CD8^+^ (%)	Pre-treatment	1.28±0.10	1.28±0.07	0.059	0.953
	Post-treatment	1.75±0.19	1.56±0.17	4.908	0.000
IgM (g/L)	Pre-treatment	1.38±0.67	1.41±0.70	0.140	0.889
	Post-treatment	2.78±0.64	2.42±0.52	2.770	0.007
IgG (g/L)	Pre-treatment	1.34±0.26	1.34±0.32	0.011	0.991
	Post-treatment	2.86±0.76	2.31±0.48	3.861	0.000
IgMA (g/L)	Pre-treatment	1.33±0.26	1.33±0.32	0.000	1.000
	Post-treatment	2.84±0.76	2.24±0.42	4.371	0.000

No evident pre-treatment differences in SGRQ scores between the two groups (*P*>0.05). By contrast, the post-treatment SGRQ scores of the two groups were significantly improved compared with those before intervention and the improvement of SGRQ scores in the observation group was higher than that of the control group, with statistically significant differences (*P*<0.05) (Table-IV).

**Table-IV:** Comparison of SGRQ Scores between the 2 Groups (*Χ̅±S*).

Group	n	Pre-treatment	Post-treatment
Observation	40	68.98±4.35	33.85±4.28
Control	40	68.08±2.96	38.15±2.95
*T*		1.082	5.231
*P*		0.283	0.000

The incidence of adverse reactions in the observation group (50%) was higher than that in the control group (40%), without statistically significant differences (*P*>0.05) (Table-V).

**Table-V:** Comparison of Adverse Reactions between the two Groups.

Group	Fever	Gastrointestinal Reactions	Peripheral Neuritis	Hepatic Impairment	Overall Incidence
Observation (n=40)	4 (10.00)	7 (17.50)	3 (7.50)	6 (15.00)	20 (50%)
Control (n=40)	5 (12.50)	4 (10.00)	2 (5.00)	5 (12.50)	16 (40%)
*χ^2^*					0.808
*P*					0.369

## DISCUSSION

In this study, the clinical efficacy and safety of lenvatinib combined with sintilimab, the immune checkpoint inhibitor, in treating advanced liver cancer was evaluated, as well as its impact on immune function of patients, revealing that the ORR in the observation group (combination therapy) was remarkably higher than that in the control group (monotherapy) (P=0.039) and the improvement in immune function indicators and SGRQ scores was more significant in the observation group than in the control group (*P*<0.05), with no statistically significant differences found in the incidence of adverse reactions between the two groups (*P*=0.369). These findings are consistent with several studies and provide new clinical evidence, particularly in terms of improving the immune function and quality of life in patients.^7^ In recent years, the incidence of primary liver cancer has been on the rise and most patients with advanced or progressive liver cancer lack surgical indications and require medication treatment. Notably, we have now entered a phase of immune combination targeted therapy. Lenvatinib is a multi-targeted tyrosine kinase inhibitor that can inhibit tumor angiogenesis and proliferation, with significant clinical efficacy;^8^,^9^ and immune checkpoint inhibitors enhance anti-tumor effects by activating T-cell immune responses.^10^

The strategy of lenvatinib combined with immunotherapy has been validated in clinical research,^11^ and the ORR of lenvatinib combined with pembrolizumab has been indicated at 36%,^12^ lower than that of the observation group in this study (72.5%), possibly due to the population in this study being primarily composed of patients with hepatitis B-associated liver cancer and the unique mechanism of action of sintilimab.^13^ Additionally, other studies have indicated that lenvatinib combined with pembrolizumab did not achieve the primary endpoint of overall survival, suggesting that the benefits of the combination regimen may be related to patient heterogeneity and the tumor microenvironment.^14^ By contrast, the response rate was higher in patients treated with lenvatinib combined with sintilimab in this study.

However, due to its small sample size and short follow-ups (median follow-up of about one year), the follow-ups should be extended in the future to further validate the conclusions of this study. Meanwhile, the efficacy of sintilimab combined with bevacizumab analogues in patients with unresectable liver cancer has been investigated, showing a better survival benefit than that of sorafenib.^15^ Despite not directly comparing survival times, this study reported a higher ORR, possibly due to the stronger anti-angiogenic effect of lenvatinib and modulation of the immune microenvironment.^16^ Moreover, lenvatinib has been reported to reduce regulatory T cells and enhance CD8+ T cell infiltration.^17^ The significant increase in the CD4+/CD8+ ratio observed in this study aligns with previous research, further supporting the immune modulation mechanism of the combination therapy.^18^

It has been theorized that lenvatinib can remodel the immune microenvironment of tumor patients, while immune checkpoint inhibitors can enhance T-cell cytotoxicity.^19^ Compared to most studies abroad that focus solely on overall response rates or median survival times, this study validates that lenvatinib combined with immune checkpoint inhibitors can synergistically enhance cellular and humoral immunity through T-cell subsets and immunoglobulin levels, thus providing clinical support for the aforementioned theory. Previous studies have paid little attention to the impact of combination therapy on the quality of life in patients.^20^

In this regard, this study demonstrated more significantly improved SGRQ scores of patients in the observation group than those in the control group (*P*<0.001), indicating that this regimen not only controls tumor progression in patients with liver cancer but may also improve their quality of life by alleviating clinical symptoms and enhancing immune function. Additionally, regional differences exist regarding the causes of liver cancer worldwide, where alcohol and metabolic-related fatty liver disease are predominant in Europe and the United States, while liver cancer primarily develops due to hepatitis B virus infection in Asia, especially in China.^21^ In this study, hepatitis B-associated liver cancer accounted for 72.5%, with the findings providing important references for combination treatment strategies in this specific population.

In this study, standardized efficacy assessments and adverse reaction grading were employed, enhancing the reliability of result comparability while focusing on the quality of life of patients, thus providing a more comprehensive reference for clinical decision-making.

### Limitations

- It involved a retrospective analysis, which may introduce selection bias and cannot control for confounding factors;
- The small sample size affects statistical power;
- A lack of long-term follow-up data due to the short median follow-up time, making it impossible to assess OS differences;
- Tumor marker levels were not monitored, which may be related to efficacy. Future randomized controlled trials with larger sample sizes and extended follow-ups are needed to observe the clinical effects and safety of combination therapy, along with enhanced biomarker monitoring to identify the optimal beneficiary population.

## CONCLUSIONS

Lenvatinib combined with immune checkpoint inhibitors can significantly increase the objective response rate, improve immune function and enhance the quality of life in patients with liver cancer without significantly increasing their adverse reactions, thereby demonstrating excellent safety.

### Authors’ Contributions:

**YH:** Study design, literature search and manuscript writing, and is responsible and accountable for the accuracy or integrity of the work.

**ZG and CL: D** ata collection, data analysis and interpretation. Critical Review.

**YZ and DZ:** Manuscript revision and validation. Critical Analysis.

All authors have read and approved the final manuscript.
